# Investigation into the Interaction between Penicillin-Resistant and Penicillin-Susceptible Gonococcal Penicillin-Binding Protein 2 and Target Phenolic Ligands through Molecular Docking Studies and Structure-Activity Relationship Analysis

**DOI:** 10.1155/2024/2585922

**Published:** 2024-06-12

**Authors:** Sinethemba Yakobi, Lindiwe Zuma, Ofentse Pooe

**Affiliations:** School of Life Sciences, Biochemistry, University of KwaZulu-Natal, Durban, South Africa

## Abstract

Gonococcal infections present a notable public health issue, and the major approach for treatment involves using *β*-lactam antibiotics that specifically target penicillin-binding protein 2 (PBP2) in *Neisseria gonorrhoeae*. This study examines the influence of flavonoids, namely, rutin, on the structural changes of PBP2 in both penicillin-resistant (FA6140) and penicillin-susceptible (FA19) strains. The research starts by clarifying the structural effects of certain mutations, such as the insertion of an aspartate residue at position 345 (Asp-345a), in the PBP2. The strain FA6140, which is resistant to penicillin, shows specific changes that lead to a decrease in penicillin binding. These mutations, namely, P551S and F504L, have a significant impact on the pace at which acylation occurs and the stability of the strain under high temperatures. Molecular docking analyses investigate the antibacterial activities of rutin and other phytocompounds, emphasising rutin's exceptional binding affinity and its potential as an inhibitor of PBP2. Quercetin and protocatechuic acid have encouraging antibacterial effectiveness, with quercetin displaying characteristics similar to those of drugs. Molecular dynamics simulations offer a detailed comprehension of the interactions between flavonoids and PBP2, highlighting rutin's exceptional antioxidant effects and strong affinity for the substrate binding site. The study's wider ramifications pertain to the pressing requirement for antiviral treatments, namely, in the context of the ongoing COVID-19 epidemic. Flavonoids have a strong affinity for binding to PBP2, indicating their potential as inhibitors to impair cell wall formation in *N. gonorrhoeae*. Ultimately, this study provides extensive knowledge on the interactions between proteins and ligands, the dynamics of the structure, and the ability of flavonoids to combat penicillin-resistant *N. gonorrhoeae* bacteria. The verified simulation outcomes establish a basis for the creation of potent inhibitors and medicinal therapies to combat infectious illnesses.

## 1. Introduction

Historically, the sexually transmitted illness gonorrhoeae, which is caused by the bacterium *Neisseria gonorrhoeae* (*N. gonorrhoeae*), was effectively treated by delivering a solitary dosage of penicillin [1–3]. However, the emergence of penicillin-resistant bacterial species has led to the exploration of other antibiotics [4]. The increase in *N. gonorrhoeae* strains exhibiting intermediate resistance to routinely prescribed antigonococcal medication poses a significant obstacle to the efficacy of treatment [5, 6]. Recent research conducted by Vincent and Jerse has revealed alarming evidence of an escalating trend in the overall resistance, which may provide difficulties in selecting appropriate treatment options [7]. The antibacterial activities of *β*-lactam antibiotics are attributed to their specific binding to penicillin-binding proteins (PBPs) [8]. Peptidoglycan biosynthesis proteins (PBPs), crucial enzymes responsible for the production of peptidoglycan in bacterial cells, may be classified into three distinct classes (A, B, and C) based on their specific structural and functional characteristics [3, 9]. Comprehending the transpeptidase function of PBPs in classes A and B is essential for promoting the creation of peptide cross-links between adjacent peptidoglycan strands. Class A PBPs possess a transglycosylase domain, which is accountable for the process of polymerisation and the formation of covalent bonds between glycan chains [8, 9]. The study conducted by Straume et al. [10] emphasises the unique characteristics of class B PBPs. PBP1, PBP2, and PBPs 3 and 4 are categorised into classes A, B, and C, respectively [11]. PBP2 enzymes have been identified as the principal target of penicillin at the minimum inhibitory concentration (MIC) in susceptible bacteria [3, 6, 12]. The appearance of penicillin-resistant bacteria, caused by PBP2 variations with an insertion of aspartic acid at the amino acid junction 345–346 (Asp-345a or D-345a), emphasises the need for alternate treatment methods [13]. The variations exhibit 4–8 substitutions in close proximity to the protein's C-terminus [14, 15]. Our work has revealed the exceptional antibacterial activity of phenolic compounds present in *Pleurotus ostreatus* mushrooms. These compounds have demonstrated significant promise in fighting drug-resistant isolates of *N. gonorrhoeae* [6, 16]. This work highlights the need to discover new bioactive chemicals that can effectively fight against antibiotic-resistant bacteria, thereby tackling the growing problem of antimicrobial resistance. Molecular dynamics simulations enhance our understanding of the interactions between phenolic compounds and PBP2. These simulations offer a comprehensive insight into the intricate molecular dynamics, highlighting the antioxidant effects and strong affinity of the compounds for the substrate binding site. The integration of molecular simulations improves the accuracy and comprehensiveness of our research, enabling a detailed investigation of the complex interactions between proteins and ligands as well as the structural changes over time. This contributes to the progress of drug development methods. Moreover, this study expands the significance of its findings to include more than just gonococcal infections. It also relates to the worldwide need for antiviral therapies during the current COVID-19 epidemic. Natural compounds' great affinity for target proteins positions them as prospective inhibitors capable of affecting cell wall formation in *N. gonorrhoeae*. This presents a flexible method in the larger context of infectious illnesses. Ultimately, this study provides a fresh viewpoint on the molecular foundations of antibiotic resistance in *N. gonorrhoeae*, presenting bioactive chemicals as promising inhibitors. By utilising the benefits of molecular simulations, our research establishes a strong basis for creating powerful inhibitors and medical treatments. This addresses the urgent issue of infectious illnesses and contributes to the worldwide fight against antibiotic resistance.

## 2. Method

### 2.1. Protein Identification

The macromolecules studied in this research were penicillin-binding protein 2 (PBP2) of the clinical penicillin-resistant mutant *N. gonorrhoea* strain FA6140 (6HZJ) [17] and PBP2 from *N. gonorrhoeae* strain FA19 (3EQU) [18]. These macromolecules were retrieved from the Research Collaboratory for Structural Bioinformatics (RCSB) Protein Data Bank (PDB), and the macromolecule studied in this research is the crystal structure of penicillin-binding protein 2.

### 2.2. Structure Validation

The structural integrity of both FA6140 and FA19 PBP2 was assessed using the use of Procheck Structure Verification Methodology. The Procheck analysis generated a Ramachandran plot that depicted the bond angles (Phi and Psi angles) of each residue, thereby verifying the credibility of the anticipated secondary structure and its 3D conformations. Furthermore, a G-factor metric was computed to assess the likelihood of error or deviation in the anticipated configuration.

### 2.3. Molecular Docking Using PyRx

#### 2.3.1. Protein Preparation

The protein complexes obtained from the RCSB PDB were processed using Discovery Studio 2021 to eliminate heteroatoms. The protein structures were modelled and then optimised for docking purposes using AutoDockTools. The resulting proteins were saved in the .pdb format.

#### 2.3.2. Ligand Preparation

Target ligands in the 3D. sdf format with high positive drug-likeness scores were retrieved from the PubChem database (https://pubchem.ncbi.nlm.nih.gov/), namely, protocatechuic acid (IUPAC name: 3,4-dihydroxybenzoic acid), p-coumaric acid (IUPAC name: (E)-3-(4-hydroxyphenyl)prop-2-enoic acid), ferulic acid (IUPAC name: (E)-3-(4-hydroxy-3-methoxyphenyl)prop-2-enoic acid), quercetin (IUPAC name: 2-(3,4-dihydroxyphenyl)-3,5,7-trihydroxychromen-4-one), and rutin (IUPAC name: 2-(3,4-dihydroxyphenyl)-5,7-dihydroxy-3-[(2S,3R,4S,5S,6R)-3,4,5-trihydroxy-6-[[(2R,3R,4R,5R,6S)-3,4,5-trihydroxy-6-methyloxan-2-yl]oxymethyl]oxan-2-yl]oxychromen-4-one). The ligands were retrieved in the .sdf format and converted to the .pdb format.

#### 2.3.3. Ligand-Protein Docking

The PyRx software was utilised to execute the ligand-protein binding procedure by employing the AutoDock Vina plugin. Upon completion of a proficient docking procedure, the protein-ligand intermolecular associations were assessed in accordance with their corresponding binding energies. The ligands' conformation was analysed through the use of Discovery Studio 2021. All molecular dynamics (MD) simulations were performed using the Desmond package.

Two independent replicates of the molecular dynamics simulations were performed for each investigated system, taking into account the intrinsic variety in molecular systems. Each replication started with unique random seed values for the initial velocities of atoms to introduce diversity in the trajectory sampling. The decision to do several replicates is motivated by the understanding that a single trajectory may not fully capture the dynamic behaviour of the system and may yield results that are heavily impacted by the beginning conditions. To assess the statistical significance of our observations, we conducted separate analyses for each replicate. This approach allows us to determine and verify the coherence of patterns and the capacity to replicate the observed chemical interactions. By including several replicates, we greatly improve the trustworthiness of our results and get a more comprehensive understanding of the dynamics of the system. While examining the binding free energy landscape, we observed similar patterns throughout the replicates, indicating the presence of consistent binding events. The RMSD profiles from the two replicates exhibited similar patterns, confirming the reproducibility of the observed conformational changes in the protein-ligand complex. Furthermore, statistical calculations were performed independently for each duplicate to determine the mean binding affinities and residence lengths. This approach enables a more accurate computation of these parameters and also streamlines the assessment of the variability associated with the simulation outcomes. The data presented in this article are the result of a comprehensive investigation of several duplicates, which improves the statistical significance and reliability of our molecular dynamics simulations. The inconsistencies identified in duplicate samples are thoroughly investigated in the subsequent sections, ensuring a comprehensive explanation of our findings.

## 3. Results

**Figure 1:**
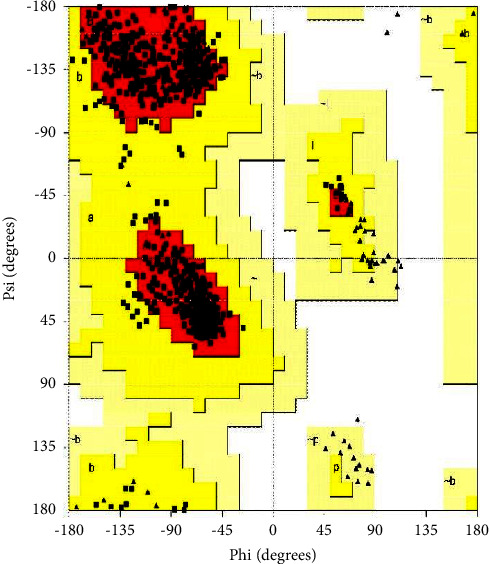
The identification of the allowed and disallowed regions of protein backbone conformation.

The structural integrity of the modelled FA6140 PBP2 was assessed using the Procheck software.  Figure 1 depicts the Ramachandran plot analysis, which showcases the statistical distribution of the combinations of the backbone dihedral angles *ϕ* and *ψ*. The Ramachandran plot delineates the permissible conformational space of the Phi/Psi torsion angles for an amino acid, X, situated within an ala-X-ala tripeptide, thus establishing the theoretically viable intervals. The plot's narrative is dichotomised into two distinct domains predicated on the presence or absence of steric hindrances amidst atoms. The region where steric collisions occur is conventionally designated as the disallowed region, while the region that lacks such collisions is commonly known as the allowed region.

**Figure 2:**
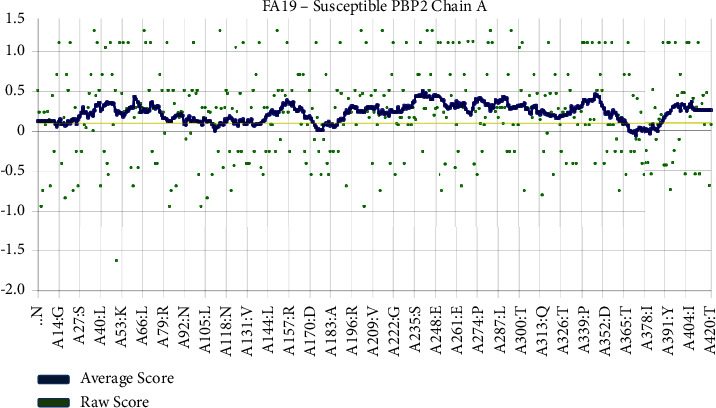
The congruence between a three-dimensional atomic model and the corresponding one-dimensional amino acid sequence of the FA19 PBP2 chain A.

The analysis of residue distribution within a protein molecule yielded significant insights pertaining to its structural and functional characteristics. The study findings indicate that a significant proportion of the protein, specifically 91.7%, was situated in the most favourable regions, namely, A, B, and L. Additionally, a smaller proportion of 8.3% was observed in other allowed regions, including a, b, l, and p. Remarkably, residues were not detected within the permissive regions (∼a, ∼b, ∼l, and ∼p) or the impermissible regions. The results indicate that the protein being studied exhibits a high degree of stability and proper folding, characterised by a compact core and limited conformational variability. Additional research is required to comprehensively comprehend the ramifications of these findings on the biological role of the protein. The analysis encompassed a total of 869 residues. All of the 715 residues analysed were found to be nonglycine and nonproline residues. Furthermore, a total of 14 terminal amino acid residues (excluding glycine and proline) were detected. The study revealed the identification of 90 glycine residues, which were denoted by triangles, and 50 proline residues. The aforementioned findings offer significant insights into the composition of the scrutinised sample. The identification of outliers in a dataset is a customary practice in statistical analysis. Outliers are data points that exhibit a substantial deviation from the remaining data. A common method for detecting outliers involves the implementation of a criterion based on a threshold of two standard deviations from the mean. Outliers in graphical data representations are commonly represented as black. The small-molecule data are represented by the solid and dashed lines, denoting the mean and standard deviation values, respectively. The Ramachandran plot analysis revealed a predominant 93.0% core region, accompanied by a minor 7.0% allowance region, while no residues were observed in the generously allowed or disallowed regions. Upon conducting an analysis of all Ramachandran, a total of three designated residues (out of 823) were detected. Additionally, on the chi1-chi2 plots, a total of seven designated residues (out of 451) were observed. The side chain parameters exhibited 5 instances of improvement, 0 instances of being situated within the interior, and 0 instances of deterioration. The analysis of residue properties revealed a maximum deviation of 11.8, 93 unfavourable contacts, 3.6 deviations in bond length and angle, and 4 cis-peptides. The G-factors exhibited a dihedral value of −0.23, a covalent value of 0.46, and an overall value of 0.05. The planar groups exhibited a 100.0% rate of conformity within the established parameters, with no instances of deviation or anomalies detected, as evidenced by the absence of any highlighted data points. The congruence between a tridimensional atomic model and its corresponding unidimensional amino acid sequence was achieved by categorizing the model's structural class based on its specific location and surrounding environment. The outcomes were then juxtaposed against the established structural benchmarks. The findings indicate that a significant proportion of residues, specifically 88.05%, exhibits an average 3D-1D score equal to or greater than 0.1.  Figure 2 illustrates that a minimum of 80% of the amino acids have obtained a score of 0.1 or higher in the 3D/1D profile. The structural integrity of the modelled FA19 PBP2 was assessed using the Procheck software.

### 3.1. Molecular Docking Analysis Using PyRx and Schrödinger

**Figure 3:**
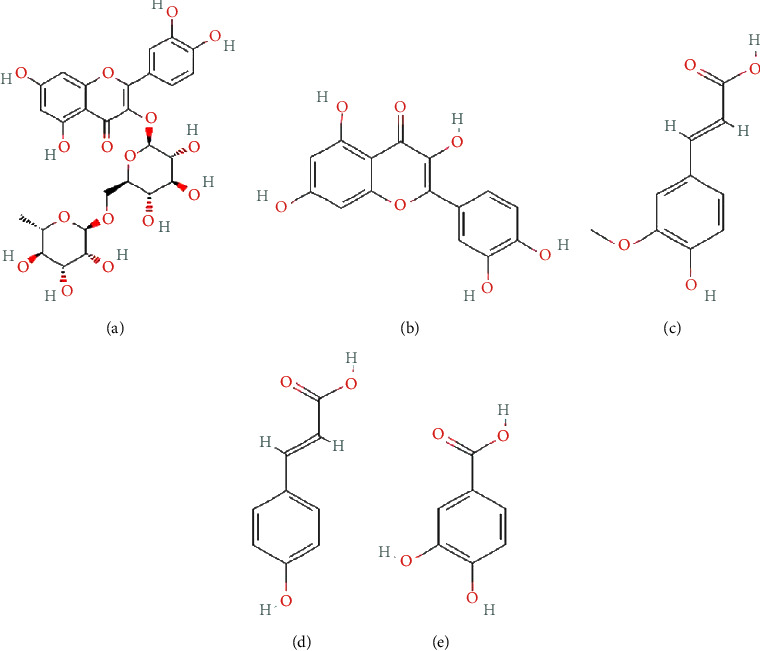
Chemical compounds identified and investigated for their antibacterial properties against penicillin-resistant *N. gonorrhoeae* isolates (PubChem). (a) Rutin. (b) Quercetin. (c) Ferulic acid. (d) 4-Hydroxycinnamic acid. (e) 3,4-Dihydroxybenzoic acid.

Phytochemicals are natural molecules found in plants that have a crucial function in safeguarding them from both living and nonliving threats. Furthermore, these chemicals possess remarkable bioactive properties that might enhance human health and general well-being. Fruits and vegetables are the main sources of phenolic compounds in the human diet. Plant-based diets are known to include the main bioactive ingredients, which are these chemicals.  Figure 3 displays a 2D chemical structure of the substance used in this experiment. The current study utilised PyRx software to conduct molecular docking studies to assess the binding affinity between certain phytochemicals and the FA6140 PBP2 model. The primary objective was to evaluate the binding energy of the mentioned chemicals.

**Figure 4:**
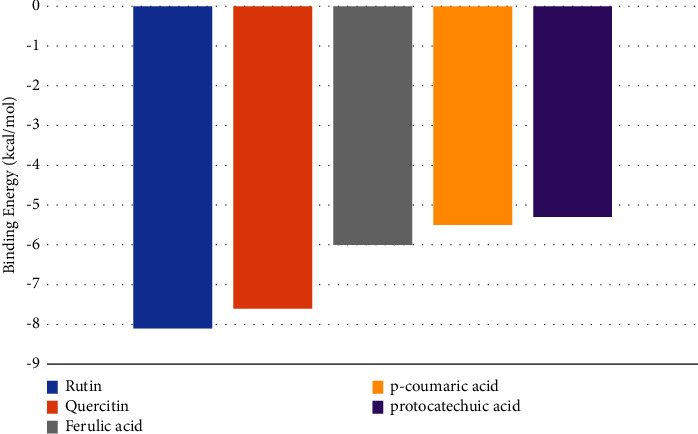
Comparative analysis of the binding energy of the various compounds.

**Figure 5:**
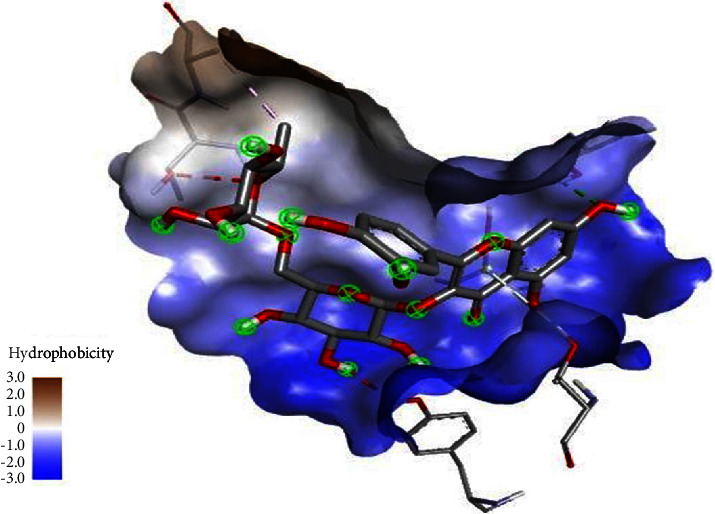
The molecular interaction between rutin and FA6140 PBP2, as well as the association of nonpolar groups or molecules in an aqueous environment.

In our investigation, we used computational techniques to replicate the process of binding and scrutinise the ensuing interaction. The present study involved the performance of molecular docking analyses on all five target compounds with the 3eqv [A] protein. The experimental findings suggest that the lowest binding energy was observed in the interaction between the 3eqv [A] FA6140 protein and rutin, followed by quercetin, ferulic acid, p-coumaric acid, and protocatechuic acid, as illustrated in Figure 4. The binding energy is subject to notable influence from the interactions that occur between ligands and the hydrophobic side chains present in proteins. It is widely recognised that hydrophobic amino acid residues demonstrate a tendency to repel water and other polar functional groups. The depicted molecular interaction between rutin and FA6140 PBP2 results in a net attraction of the ligand's nonpolar groups. This phenomenon involves the association of nonpolar groups or molecules in an aqueous environment, as illustrated in Figure 5. The results indicate that the compound rutin engages in noncovalent interactions with FA6140 PBP2, involving hydrogen bonding and hydrophobic interactions. Additionally, the research demonstrates that hydrophobic groups or molecules have a tendency to aggregate in an aqueous medium as a result of the hydrophobic effect.

**Figure 6:**
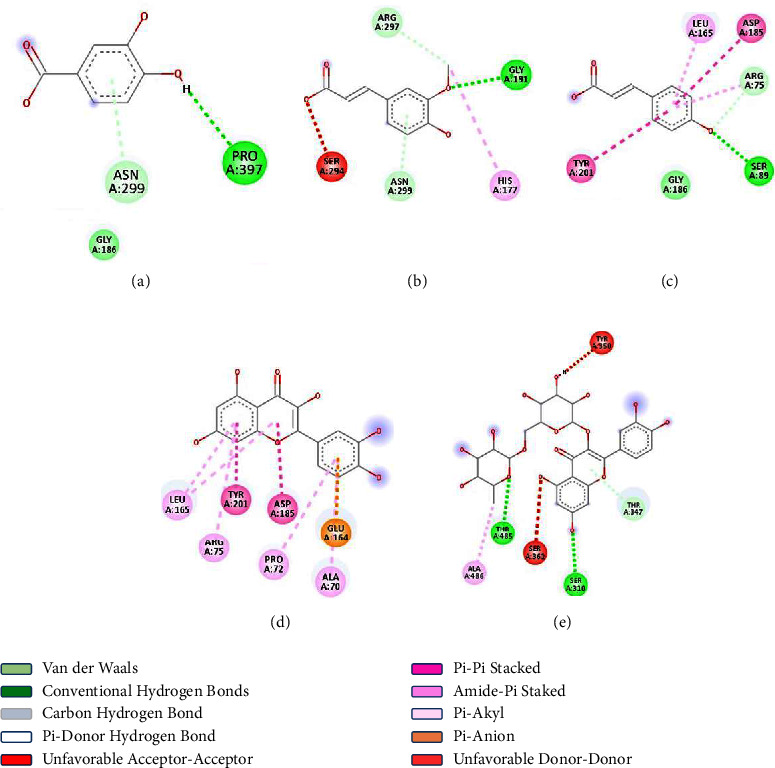
A graphical representation of the interactions between the target protein and the selected ligands in two dimensions. (a) Protocatechuic acid. (b) Ferulic acid. (c) *p*-Coumaric acid. (d) Quercetin. (e) Rutin.

In the context of the interaction between quercetin and FA6140 PBP2, the charge distribution within the molecule or complex system can be estimated or calculated. The interpolated charge may be associated with the distribution of charges on the atoms within quercetin during the binding process. Having a clear grasp of the charge distribution is essential in order to fully grasp the electrostatic interactions between the ligand (quercetin) and the protein (FA6140 PBP2). We have extensive knowledge and expertise in the field of bioinformatics. The interaction between quercetin and FA6140 PBP2 is characterised by a robust binding affinity and specific interactions with crucial amino acids. The results obtained from the docking analysis confirm the molecular basis of the interaction and conformation of the specified phytochemicals with the binding sites of FA6140 PBP2. Furthermore, the results indicate that the phytochemicals possess the capacity to establish numerous hydrogen bonds and noncovalent interactions with the crucial functional residues of the protein under investigation. Based on the empirical evidence, rutin demonstrates the most significant binding affinity towards the target protein when compared to the other compounds. The molecule exhibits the capacity to form strong hydrogen bonds with essential functional amino acids, such as THR-485, SER-310, and a Pi-donor hydrogen bond with THR-347, leading to a docking score of −8.1 kcal/mol. The compound in question exhibited an interaction with the ALA-485 amino acid, which is classified as an alkyl interaction. Furthermore, it demonstrates unfavourable donor-donor and acceptor-acceptor interactions with TYR-350 and SER-362, respectively, as illustrated in Figure 6. Quercetin displayed significant binding affinity to the target protein and formed strong interactions with the crucial functional amino acids, such as Pi-alkyl bonds at LER-165, ARG-75, PRO-72, and ALA-70; van der Waals bonds at GLY-186; Pi-anion bonds at GLU-164; and Pi-Pi stacked bonds and amine-Pi stacked bonds at TYR-201 and ASP-185, correspondingly. The thermodynamic stability of the interaction was assessed by computing the docking score, which was found to be −7.6 kcal/mol. Furthermore, Figure 6 provides evidence of unfavourable interactions between the donor and acceptor moieties with TYR-350 and SER-362, respectively.

The results indicate that hydrogen bonds were formed between ferulic acid and PRO-191 and ASN-299, while a Pi-alkyl bond was formed with HIS-177. An unfavourable acceptor-acceptor bond was observed between ferulic acid and SER-294, and a Pi-donor hydrogen bond was formed with ARG-297. The binding affinity between ferulic acid and the aforementioned amino acids was calculated to be −6.0 kcal/mol. p-coumaric acid exhibited a binding affinity of −5.5 kcal/mol and engaged in hydrogen bonding interactions with the protein at SER-89 and ARG-75. The compound exhibited interactions with LEU-165 through a Pi-alkyl bond, ASP-185 through an amine-Pi stacked bond, GLY-186 through a van der Waals bond, and TYR-201 through a Pi-Pi stacked bond. Finally, the interaction between protocatechuic acid and PRO-397 was established through conventional hydrogen bonding and ASN-299 Pi-donor hydrogen bonding, exhibiting a binding affinity of −5.3 kcal/mol.

The outcomes derived from the docking analysis validate the molecular underpinnings of the interplay and configuration of the designated phytochemicals with the binding locales of FA19 PBP2. The docking study's results are displayed in Table 1, which aimed to anticipate the binding affinity and orientation of the compounds in the protein's active site. Additionally, the findings suggest that the phytochemicals exhibit the ability to form multiple hydrogen bonds and noncovalent interactions with the essential functional residues of the protein being studied. The results obtained from empirical studies indicate that rutin exhibits the highest binding affinity towards the target protein in comparison with the other compounds. The molecular entity demonstrates the ability to establish robust hydrogen bonding interactions with critical functional amino acid residues, namely, THR-485, SER-310, GLY-481, and GLY-482, resulting in a docking score of −8.1 kcal/mol. Rutin demonstrated an interaction with the amino acid THR-347, which is classified as a carbon-hydrogen bond. Moreover, it exhibits an alkyl linkage with ALA-496, as depicted in Table 1. Furthermore, the compound quercetin exhibited noteworthy binding affinity towards the designated protein and established robust interactions with the essential functional amino acids, including conventional hydrogen bonds at LEU-165, SER-89, and TYR-201; van der Waals bonds at GLY-186; Pi-anion bonds at GLU-164; Pi-Pi stacked bonds and amine-Pi Stacked bonds at TYR-201 and ASP-185, respectively; and Pi-alkyl bonds with ARG-75. The thermodynamic stability of the interaction was evaluated through the calculation of the docking score, yielding a value of −7.8 kcal/mol, as presented in Table 1.

### 3.2. Molecular Dynamics Simulations

#### 3.2.1. Rutin-Penicillin-Resistant PBP2

**Figure 7:**
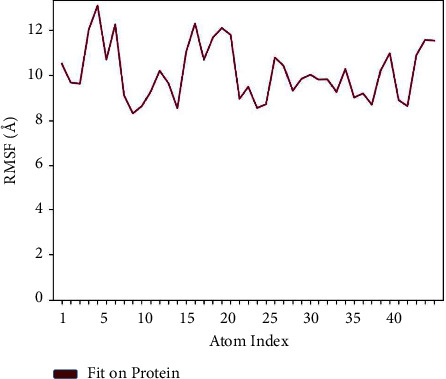
Atomic fluctuations in protein and ligand: RMSF analysis of molecular dynamics simulation.

An extensive investigation was conducted on rutin-penicillin-resistant PBP2, which included a thorough assessment of molecular dynamics (MD) simulations using the Desmond package. The data display the results of root-mean-square fluctuation (RMSF) calculations for various atoms in a molecular system. These calculations are frequently employed in molecular dynamics simulations to assess the flexibility or mobility of atoms in a protein or ligand over a given period of time. The data include the atom index or identifier within the system, the residue name in the Protein Data Bank (PDB) format, and the RMSF value relative to the protein. The statement describes the representation of the protein's atom fluctuation or flexibility, as well as the root-mean-square fluctuation (RMSF) value in relation to the ligand. The “wrt_Protein” values represent the variability of individual atoms within the protein structure. Greater values indicate more flexibility or mobility of the atoms in question. The “wrt_Ligand” values represent the variability of individual atoms within the ligand. Likewise, elevated values indicate more flexibility or mobility of the atoms in the ligand. Examining atom# 5, it possesses a “wrt_protein” value of 13.084 and a “wrt_ligand” value of 1.527. These findings indicate that atom #5 in the protein exhibits a degree of flexibility, and there is a moderate level of variability in the ligand as well. Generally, the RMSF values offer valuable information on the movement of atoms in both the protein and the ligand. This information is essential for comprehending the stability and interactions within the molecular system, as seen in Figure 7.

**Figure 8:**
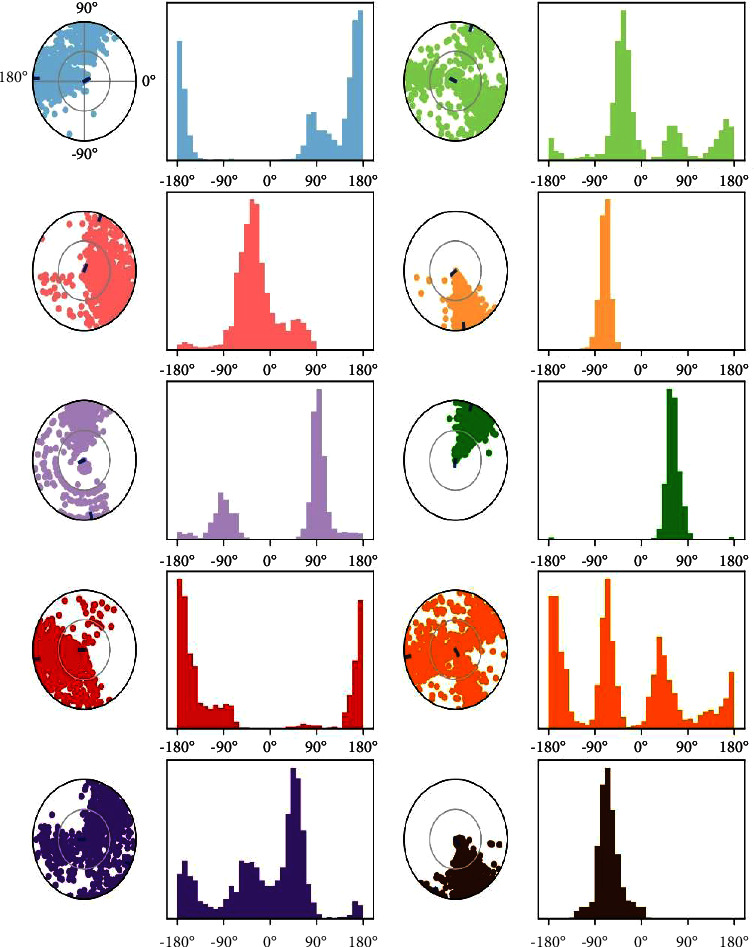
Torsion angle profiles in molecular dynamics simulation.

The torsion angles, also known as dihedral angles, in a molecular system are represented by data that are determined by the atomIDs in the system. These angles quantify the rotation between the planes of four successive atoms in the molecular structure. The dihedral angle is determined by four atomIDs, namely, ai, aj, ak, and al. Every row in the data corresponds to a distinct frame or time point in a molecular dynamics simulation. The columns display the numerical values of the dihedral angles (measured in degrees) for each specified torsion (dihedral) in the system. The numbers in each cell of the table represent the precise angle (measured in degrees) for the matching dihedral at a certain frame. Analysing a particular entry (e.g., entry at Frame 0, dihed1), the dihedral angle formed by atoms with the IDs 27, 24, 10, and 64 measures 24.876 degrees at Frame 0. The dihedral angles exhibit variability across successive frames, suggesting the dynamic character of the molecular system, as seen in Figure 8. The system investigates various torsional conformations, as indicated by the varying values of the dihedral angles. Positive and negative values signify the direction of rotation at these angles. The ligand torsion graphic provides a concise representation of the changes in conformation of each rotatable bond (RB) in the ligand during the whole simulation track (0.00 to 100.00 nsec). The upper panel displays a two-dimensional diagram of a ligand, with rotatable bonds highlighted using different colours. A dial plot and bar plots of the same colour are provided for each rotatable bond torsion. Dial (or radial) graphs depict the configuration of the torsion angle across the duration of the simulation. The simulation starts at the central point of the radial plot, and the temporal progression is depicted in a radial outward manner. The bar plots provide a concise representation of the data from the dial plots, displaying the probability density of the torsion. If data on torsional potential are provided, the figure will further display the potential of the rotatable bond by aggregating the potential of the corresponding torsions. The potential values are displayed on the *Y*-axis of the chart, positioned to the left, and are denoted in units of kcal/mol. Examining the correlation between the histogram and torsion potential might provide valuable information about the structural stress experienced by the ligand in order to retain its shape while attached to a protein.

The L_Properties are derived from a molecular dynamics simulation or similar computational research, and they represent distinct properties of a molecular system at different frames or time steps. The simulation's frame or time step, which quantifies the average deviation of atomic locations between the current frame and a reference structure, provides insight into the extent of structural changes occurring during the simulation. The radius of gyration provides a quantitative assessment of the molecular structure's compactness, while the intramolecular hydrogen bonds indicate the quantity of hydrogen bonds produced within the molecule. The molecular surface area represents the overall surface area of the molecule, whereas the solvent-accessible surface area specifically indicates the portion of the molecule's surface that can be reached by solvent molecules. On the other hand, the polar surface area refers to the surface area of the molecule that is occupied by polar atoms. Our simulation revealed that the system experiences structural modifications, characterised by an elevation in RMSD and variations in other characteristics. Notably, there is a large increase in RMSD, suggesting a more pronounced divergence from the initial structure. Some frames exhibit stabilisation, characterised by a generally constant root-mean-square deviation (RMSD) and other properties. Other frames, however, demonstrate oscillations in the system, but the properties do not display any major patterns. These interpretations are derived from overarching patterns, and a more comprehensive examination may be necessary to formulate precise conclusions on the behaviour of the molecular system during the simulation.

**Figure 9:**
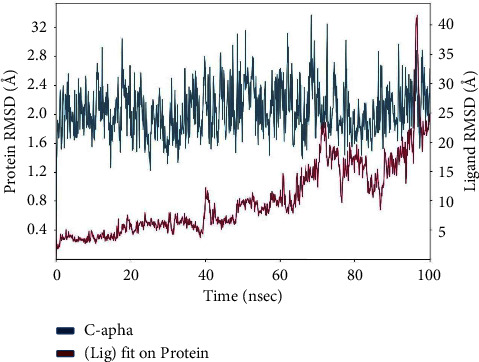
Protein-ligand complex RMSD over time: exploring structural dynamics of rutin-penicillin-resistant PBP2 complex.

Figure 9 illustrates the progressive changes in the root-mean-square deviation (RMSD) of a protein, as indicated on the left *Y*-axis. The protein frames are initially aligned with the reference frame backbone, and then, the root-mean-square deviation (RMSD) is computed using the selected atoms. Tracking the root-mean-square deviation (RMSD) of the protein can provide valuable information about its structural conformation during the simulation. RMSD analysis may determine if the simulation has reached equilibrium by examining the fluctuations near the conclusion of the simulation, which should be close to the average thermal structure. Small, globular proteins may tolerate changes in the range of 1–3 Å without any issues. Significant alterations beyond the aforementioned magnitude suggest that the protein is experiencing a substantial structural transformation throughout the simulation. Additionally, it is crucial for your simulation to achieve convergence when the root-mean-square deviation (RMSD) values reach a stable and unchanging number. If the root-mean-square deviation (RMSD) of the protein exhibits a consistent increase or decrease during the simulation, it indicates that the system has not reached equilibrium. Consequently, the simulation duration may not be sufficient for rigorous analysis. The ligand RMSD (shown on the right *Y*-axis) quantifies the stability of the ligand in relation to the protein and its binding pocket. The graphic above displays the RMSD (root-mean-square deviation) of a ligand in the protein-ligand complex. The alignment is performed by aligning the protein backbone of the complex to a reference structure, and then, the RMSD is calculated for the ligand's heavy atoms. If the measured values are considerably higher than the root-mean-square deviation (RMSD) of the protein, it is probable that the ligand has dispersed from its original binding site.

**Figure 10:**
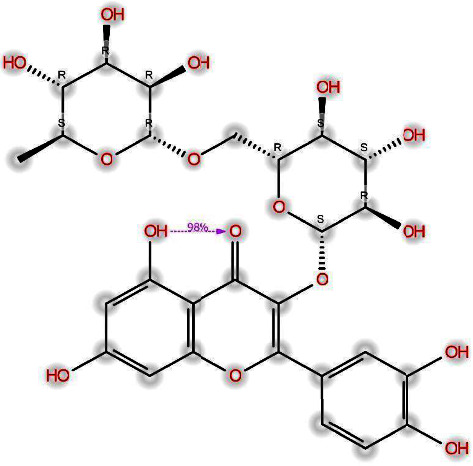
Exploring ligand-protein interactions: contact analysis between rutin and penicillin-binding protein 2 in *Neisseria gonorrhoeae.*

**Figure 11:**
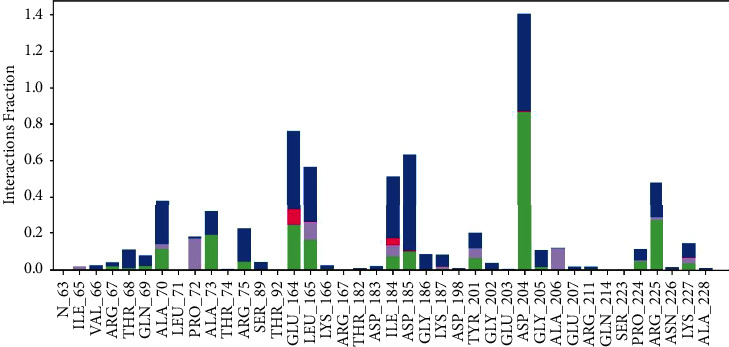
Protein-ligand interaction dynamics: categorised contacts over the trajectory.

Figure 10 illustrates the specific interactions between ligand atoms and protein residues. Interactions that take place for more than 30.0% of the simulation duration in the chosen trajectory (0.00 through 100.00 nsec) are displayed. Interactions exceeding 100% can occur when certain residues form several interactions of the same kind with a single ligand atom. As an example, the ARG side chain possesses four H-bond donors, all of which may form hydrogen bonds with a single H-bond acceptor. The findings demonstrate that the ligand's interactions with proteins were consistently observed and categorised into certain kinds throughout the simulation. The classification comprises hydrogen bonds, hydrophobic interactions, ionic interactions, and water bridges. Each of these interaction types possesses more precise subtypes, offering intricate understanding of the characteristics of the interactions. The graph displayed in Figure 11 provides a concise summary of the distribution of protein-ligand interactions across the simulation trajectory. The interactions are standardised, enabling a precise comparison of their rates. For example, a number of 0.7 indicates that the particular contact was sustained for 70% of the duration of the experiment. It is worth noting that values greater than 1.0 can occur, suggesting that specific protein residues may have numerous interactions of the same kind with the ligand. This extensive analysis enables researchers to comprehend the frequency and consistency of various interactions between the protein and ligand during the simulation, offering vital insights into the dynamics of the molecular complex.

#### 3.2.2. Rutin-Penicillin-Susceptible PBP2

An in-depth investigation of molecular dynamics (MD) simulations using the Desmond package was conducted to examine rutin-penicillin-susceptible PBP2. For atoms 1 to 5, there is a general increase in the values of “wrt_Protein,” showing a consistent trend in the measured characteristic with respect to the protein. Atoms 6 to 15 have diverse values for both “wrt_Protein” and “wrt_Ligand,” indicating probable disparities in the behaviour of these atoms in relation to both the protein and ligand. Atoms 16 to 43 display a combination of values, with variations observed in both “wrt_Protein” and “wrt_Ligand.” The root-mean-square fluctuation (RMSF) is a valuable tool for quantifying localised variations along the protein chain.

**Figure 12:**
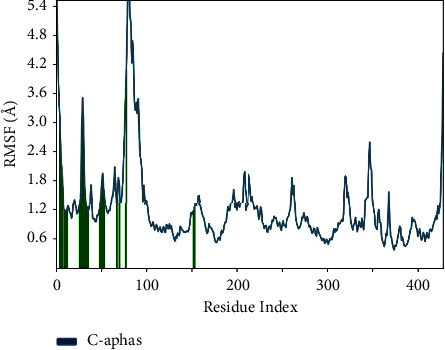
Protein dynamics: root-mean-square fluctuation (RMSF) analysis.

**Figure 13:**
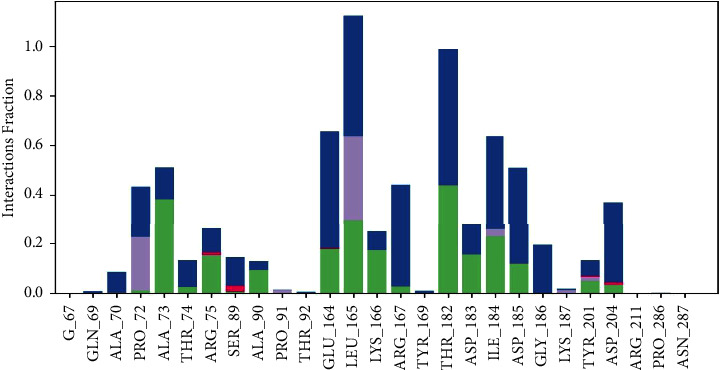
Protein-ligand contacts: categorisation and dynamics of interactions throughout the simulation.

In Figure 12, the graphic demonstrates the dynamic behaviour of the protein during the experiment, with peaks denoting areas experiencing the most volatility. Generally, the N- and C-terminal tails display more significant changes compared to other areas. In contrast, secondary structural components such as alpha helices and beta strands exhibit more stiffness in comparison with unstructured regions, leading to less fluctuation, particularly when compared to loop regions. The ligand contacts section offers significant insights into the protein residues that interact with the ligand. These interactions are graphically shown by vertical bars coloured in green. Understanding the particular residues involved in ligand interactions enhances our overall comprehension of the dynamics of protein-ligand binding during the simulation.  Figure 13 depicts the essential simulation feature of monitoring protein interactions with the ligand. The graphic presented above classifies and summarises these interactions based on their kind. The interactions, also known as “contacts,” are categorised into four primary types: hydrogen bonds, hydrophobic, ionic, and water bridges. Each type has further subtypes that are described in the “Simulation Interactions Diagram” panel. Hydrogen bonds have a crucial role in the binding of ligands, affecting the specificity of drugs, their metabolism, and their adsorption. There are four subtypes under this category: backbone acceptor, backbone donor, side chain acceptor, and side chain donor. The protein-ligand H-bond is evaluated based on precise distance and angle measurements, which provide a thorough insight into the dynamics of the interaction. This category has three subtypes: *π*-cation, *π*-*π*, and other nonspecific interactions. The geometric criteria for each subtype are defined by the proximity and orientation requirements, usually requiring a hydrophobic amino acid and an aromatic or aliphatic group on the ligand. Ionic interactions, also known as polar interactions, take place between atoms having opposing charges that are located within a specific distance from each other. There are two distinct kinds, which may be differentiated based on whether the contact is facilitated by the protein backbone or the side chains. Furthermore, the monitoring of protein-metal-ligand interactions includes the observation of a metal ion that is coordinated within a particular distance of protein and ligand heavy atoms. Water bridges refer to the hydrogen-bonded connections facilitated by water molecules between the protein and ligand. The geometric criteria for these interactions involve more lenient parameters in comparison with conventional H-bond definitions, ensuring a subtle and detailed evaluation. The stacked bar charts in the graphic are normalised based on the trajectory, representing the proportion of simulation time that each given interaction type is sustained. Values above 1.0 can occur, indicating cases when a protein residue establishes several interactions of the same subtype with the ligand. This research provides a thorough knowledge of the complex network of interactions that control the protein-ligand complex. It offers useful insights for drug design and understanding structural dynamics.

**Figure 14:**
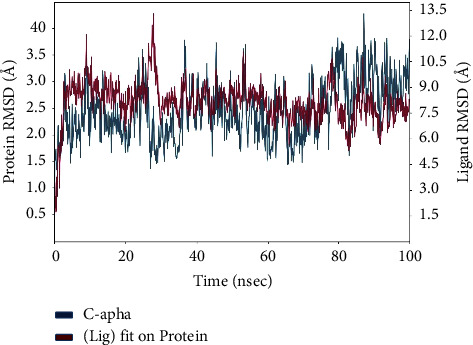
Structural dynamics analysis: RMSD evolution of the protein and ligand in the simulation.

The graphic depicts the progression of the root-mean-square deviation (RMSD) of the protein and ligand during the simulation, illustrated in Figure 14. The *Y*-axis on the left side depicts the root-mean-square deviation (RMSD) values of the protein, which indicate the degree of structural differences from the reference frame backbone. Tracking the root-mean-square deviation (RMSD) of proteins allows for the examination of the changes in structural conformation during the course of the simulation. The presence of fluctuations around the thermal average structure at the end of the simulation indicates that equilibration is occurring. Small, globular proteins are deemed acceptable if they exhibit changes within the range of 1–3 Å. Nevertheless, more substantial modifications may suggest noteworthy conformational shifts.

The *Y*-axis on the right side of the graph represents ligand RMSD, which indicates the stability of the ligand in relation to the protein and its binding pocket. The “Lig fit Prot” values represent the root-mean-square deviation (RMSD) of the ligand when it is aligned with the protein backbone of the reference frame. If the ligand RMSD values are significantly greater than the protein RMSD, it indicates the possible diffusion of the ligand from its original binding site. This observation is essential for evaluating the stability of the ligand within the binding pocket and comprehending its behaviour with respect to the protein during the simulation.

## 4. Discussion

When dealing with gonococcal infections, the main objective of *β*-lactam antibiotics is to target penicillin-binding protein 2 (PBP2) in *N. gonorrhoeae* with precision. Certain strains of *N. gonorrhoeae*, such as FA6140, exhibit the addition of an aspartate residue following the 345th position in the PBP2. According to a source, there are 4–8 additional changes that occur alongside this insertion, referred to as Asp-345a. Nevertheless, the impact of these mutations on the protein's structural integrity remains uncertain. I used the crystal structure of PBP2 from the penicillin-resistant strain FA6140 [15] to perform the analysis on the target protein. This strain shows four mutations in the C-terminal region of the protein, resulting in a notable fivefold reduction in the rate of penicillin binding compared to the standard type. Through extensive kinetic investigations, it has been discovered that the acylation rate experiences a significant decrease due to two specific mutations, namely, P551S and F504L [13]. These mutations also lead to a reduction in the enzyme's thermal stability, as evident from the melting curves. Furthermore, previous studies have highlighted the impressive antibacterial and antifungal properties of rutin, enabling it to effectively combat a wide range of harmful microorganisms. By exploring molecular interactions and binding affinities, our aim is to uncover promising inhibitors that can effectively hinder the activity of penicillin-binding protein 2. This research aligns with the ongoing efforts to develop bioactive inhibitors that are both safe and efficient for targeting viral proteins associated with COVID-19 [19]. Additional investigation has unveiled the remarkable ability of rutin to efficiently attach to the PBP2 of FA19 *Neisseria gonorrhoeae*, a vital catalyst in the creation of the microbial cellular barrier. Prior research has shown the efficacy of rutin in fighting gram-negative bacteria, indicating a possible mechanism that involves its interaction with PBP2 [20, 21]. By studying the FA6140 and FA19 PBP2, one can gain a deep understanding of how rutin affects important proteins in both penicillin-resistant and penicillin-susceptible strains of *N. gonorrhoeae*. This study uncovers the complex interactions between these proteins and rutin. Having a deep understanding of the intricate molecular mechanisms involved in antibiotic resistance is of utmost importance. These specific mutations have been discovered to interfere with the active site, resulting in challenges for antibiotics to bind or for essential conformational changes to take place during *β*-lactam antibiotic acylation. Furthermore, certain phenolic compounds have demonstrated exceptional antibacterial activity, in addition to their widely recognised antioxidant properties [15]. A selection of five compounds was made to investigate the interactions between the penicillin-resistant FA6140 PBP2 and certain phytocompounds with antibacterial properties. The selection of these compounds was based on their prior in vitro antibacterial activity. The study aimed to gain insights into the antibacterial mechanisms of these compounds, which have been observed to affect the permeability of cell membranes, interact with enzymes through hydrogen bonding to influence intracellular functions, and modify the rigidity of cell walls. These interactions with the cell membrane can result in damage to its integrity, as shown in previous studies [6, 21–23]. Advanced analytical techniques were employed to simulate the binding process and evaluate interactions. Extensive molecular docking analyses were conducted on all five target compounds against the FA6140 PBP2 enzyme's 3eqv [A]. According to the experimental findings, it was noticed that rutin exhibited the lowest energy consumption during the binding process. After that, quercetin, ferulic acid, *p*-coumaric acid, and protocatechuic acid were observed in that specific sequence. These findings provide further support for previous in vitro research, suggesting that specific flavonoids, such as quercetin and rutin, demonstrate superior antibacterial efficacy in comparison with other compounds. Rutin, with its molecular formula C_27_H_30_O_16_, exhibits remarkable antioxidant properties, effectively diminishing various oxidising species such as superoxide, peroxyl, and hydroxyl radicals. In addition, it shows noteworthy interactions with the FA6140 PBP2 enzyme. This comprehensive analysis deepens our comprehension of the antibacterial properties of these compounds, particularly with regard to the penicillin-resistant FA6140 PBP2 enzyme. Moreover, these compounds exhibit pharmacological properties, including anticancer, antibacterial, and anti-inflammatory effects. Our research indicates that both quercetin and rutin have the ability to bind to the substrate binding sites of both FA19 and FA6140 PBP2 enzymes. The FA6140 PBP2 binding site shows a significant affinity for rutin, as indicated by its remarkable binding energy of −8.1 kcal/mol. Despite its strong binding affinity, rutin does not possess drug-like properties, according to Lipinski's rule. However, quercetin, with a log Po/w of 1.63 and no drug-likeness violations, seems to be a more encouraging prospect. In previous studies, it was noted that caulerpin adheres to Lipinski's rule and has a satisfactory ADMET profile, indicating its potential as a safe drug against the SARS-CoV-2-3CL main protease. There is potential for this natural compound to be utilised in drug development in the future [24]. In line with this investigation, our research showcases the successful blocking of the FA6140 PBP2's main function by these plant compounds, leading to the prevention of the formation of penicillin-resistant *N. gonorrhoeae* bacteria. Quercetin exhibits a significant affinity of −7.6 kcal/mol for FA6140 PBP2, suggesting a strong attraction. The primary factors that govern this interaction are the energies associated with van der Waals and electrostatic forces. Acquiring three-dimensional structures through crystallography can be quite a complex process due to various factors such as crystal packing and static disorder. Building upon our previous laboratory research, we discovered that flavonoids such as quercetin and rutin have shown improved effectiveness. In our latest study, we found that protocatechuic acid exhibited a binding energy of −6.4 kcal/mol, indicating a strong affinity for the FA19 PBP2 binding site. It seems that protocatechuic acid has the ability to disrupt the main function of the FA19 PBP2, which could hinder the growth of penicillin-resistant gonorrhoea in cells. Studies have shown that rutin, with its remarkable antioxidant properties, has the ability to significantly mitigate the effects of different oxidising agents. In addition, it demonstrates a range of pharmacological activities, such as antineoplastic, antibacterial, and anti-inflammatory properties. Upon analysing the interaction with FA19 PBP2, it was observed that both rutin and quercetin exhibited a significant affinity towards the substrate binding site. However, rutin displayed a higher binding energy of −8.1 kcal/mol. Stable complexes were observed in molecular dynamics simulations with FA6140 PBP2. After conducting a thorough analysis, it was noticed that the presence of rutin resulted in a reduction in the solvent-accessible surface area. There appears to be a favourable alignment between the ligand and the binding pocket. Flavonoids possess remarkable binding capabilities that surpass those of other plant compounds, making them highly intriguing substances for optimising leads and driving pharmaceutical development forward. As part of a broader context, this study emphasises the significance of creating medications for infectious diseases, with a particular focus on the urgent need for antiviral therapies that can combat the SARS-CoV-2 virus amidst the current pandemic [25, 26]. Flavonoids have a strong binding affinity to the central functional units of FA6140 PBP2 and FA19 PBP2. Blind docking is a computational method that enables ligands to autonomously navigate the whole surface of a target protein without any prior knowledge of the binding location. This approach enhances our comprehension of the binding location and pharmacophoric interactions through many means. Blind docking is a method that may anticipate probable binding sites on a protein, uncovering specific areas where ligands exhibit a strong attraction. This is particularly advantageous in cases where the specific binding site is unidentified or when there are several putative binding pockets. Ligands have the ability to interact with many parts of the protein surface, rather than being limited to specific binding sites. This enables a thorough examination of the whole protein surface. This aids in comprehending the presence of alternate binding areas outside established locations. Blind docking can reveal allosteric binding sites, which are locations that are far from the active site and can influence the activity of proteins. These websites are essential for comprehending regulatory systems. Ligands have the ability to assume diverse conformations during blind docking, which allows for a better understanding of their flexibility and the manner in which they interact with different areas of the protein. Understanding pharmacophoric interactions is essential. Blind docking enables the anticipation of unexplored connections between ligands and proteins, which might possibly unveil new binding locations or alternate binding mechanisms. Blind docking facilitated the identification of pharmacophoric characteristics, including hydrogen bonding and hydrophobic interactions, on the protein surface by analysing the docked poses of ligands. This knowledge facilitates comprehension of the essential characteristics necessary for binding. Blind docking can function as a means of confirming the accuracy of experimental results. Aligning the anticipated binding locations with existing experimental data enhances the reliability of the computational predictions. Blind docking experiments can uncover the impact of protein flexibility on ligand binding. Proteins have dynamic characteristics, and blind docking simulations can effectively reflect the protein's capacity to adapt to various ligand conformations. Blind docking is a supplementary technique to the existing docking approaches that prioritise certain binding sites. Blind docking allows for a comprehensive examination, whereas focused docking studies provide specific and detailed information about known binding areas. The simultaneous use of both methodologies enhances our comprehensive comprehension of ligand-protein interactions. The analysis of the interaction between rutin- and penicillin-suppressed PBP2, as well as the simulation of rutin- and penicillin-resistant PBP2, produced promising results. These results indicate a successful and accurate simulation. The RMSD plot showed fluctuations within the range of 1–3 Å, suggesting that there were acceptable variations for small, globular proteins. The simulation successfully reached convergence, displaying consistent RMSD values towards the end, indicating equilibration. The RMSD fluctuations remained within 1.5 Å throughout the simulation, indicating a well-equilibrated system. Peaks of ligand RMSD were observed, which corresponded to areas of significant protein fluctuation, such as the N- and C-terminal tails. The ligand RMSD displayed periodic peaks that coincided with protein fluctuations, yet it consistently stayed below 2.0 Å. This suggests that the ligand remains stable within the binding pocket. The plot clearly highlighted the protein residues that interacted with the ligand, and we observed that structural changes were gained through the distribution of secondary structure elements (SSEs). Prominent ligand contacts were observed, with significant interactions in important secondary structure elements, demonstrating a strong binding pattern. The ligand RMSF exhibited localised fluctuations, highlighting specific atom-level interactions that contribute to stable binding events. The maintenance of hydrogen bonds and hydrophobic interactions was consistent throughout, as indicated by the normalised frequencies of binding. The timeline representation provided a concise overview of the specific contacts, while the schematic diagrams emphasised the interactions that occurred for a significant portion of the simulation time. The timeline representation revealed sustained contacts, while the schematic diagrams highlighted important interactions, giving a clear picture of the stable binding interface. The ligand torsions displayed smooth transitions, and the radial plots indicated minimal conformational strain, thus confirming the stability of the ligand in the protein-bound state. The simulation showcased promising attributes, including smooth RMSD, stable ligand behaviour, consistent interactions, and minimal conformational strain. These findings collectively suggest a dependable portrayal of the dynamics of the protein-ligand complex. In summary, these analyses offer a thorough grasp of the dynamic behaviour of the protein-ligand complex, revealing insights into structural changes, stability, and interactions during the simulation.

## 5. Conclusion

We are investigating the antibacterial effects of rutin and other phytocompounds on two strains of *N. gonorrhoeae* in our research. The binding affinity of rutin is highlighted through molecular docking analyses, suggesting its potential as an inhibitor of PBP2. Additional research on quercetin and protocatechuic acid highlights their potential as effective antibacterial agents, particularly quercetin, which exhibits properties similar to those of pharmaceutical drugs. The molecular dynamics simulations offer a thorough comprehension of the interactions between flavonoids and PBP2. The affinity of rutin for the substrate binding site indicates its potential to inhibit penicillin-resistant *N. gonorrhoeae*, thanks to its impressive antioxidant properties. Quercetin shows great potential as a promising candidate with strong attraction and drug-like properties. The study's importance reaches beyond the scope of infectious disease medications, highlighting the crucial role of antiviral therapies, particularly in the midst of the ongoing COVID-19 pandemic. Flavonoids have strong binding affinities to PBP2, indicating their potential as inhibitors to disrupt cell wall synthesis in *N. gonorrhoeae*. Our research offers a thorough and insightful analysis of the protein-ligand interactions, revealing the antibacterial properties of flavonoids against penicillin-resistant *N. gonorrhoeae*. The simulation results indicating stability and accuracy provide valuable insights for the development of effective inhibitors and the advancement of pharmaceutical interventions against infectious diseases.

## Figures and Tables

**Table 1:** The molecular docking of various target compounds with the 3equ [A] protein.

Compound name	PubChem CID	Docking score (kcal/mol)	Hydrogen bond interaction	Other interactions
Protocatechuic acid	72	−6.4	LEU-165, ASP-204, ALA-73, TYR-201	TYR-75, ARG-75
p-Coumaric acid	637542	−5.7	SER–89, ARG–75	LEU–165, ASP–185, GLY–186, TYR–201
Ferulic acid	445858	−5.9	TYP-201	ARG-75, LEU-165, AGR167
Quercetin	5280343	−7.8	LEU-165, SER-89, TYR-201	GLY-186; GLU-164; TYR-201, ASP-185, ARG-75
Rutin	5280805	−8.1	THR-485, SER-310, GLY-481, GLY-482	THR-347, ALA-496

## Data Availability

All data generated or analysed during this study are included in this published article.
